# Evaluation of Splenic Volume and Surface Area According to Age and Gender: A Three-Dimensional Slicer Computed Tomography Study—Imaging

**DOI:** 10.3390/diagnostics16091406

**Published:** 2026-05-06

**Authors:** Busra Pirinc, Ayse Gamze Ozcan, Ekrem Solmaz, Betul Sevindik, Emine Uysal, Zeliha Fazliogullari

**Affiliations:** 1 Faculty of Medicine, Department of Anatomy, Selcuk University, 42130 Konya, Türkiye; draysegamzeozcan@gmail.com (A.G.O.); drsolmazekrem@gmail.com (E.S.); drbetulsevindik@gmail.com (B.S.); z_topal@yahoo.com (Z.F.); 2 Faculty of Medicine, Department of Radiology, Selcuk University, 42130 Konya, Türkiye; druysalemine@gmail.com

**Keywords:** volume, area, spleen, anatomy, three-dimensional imaging, computed tomography

## Abstract

**Background:** The spleen is an important organ in the evaluation of systemic diseases due to its hematologic and immunologic functions. Changes in splenic volume and surface area have diagnostic significance in various clinical conditions. This study aimed to evaluate age- and sex-related changes in splenic volume and surface area in an adult population. **Material:** A total of 304 abdominal CT scans (153 females, 151 males) were retrospectively evaluated. Participants aged 19–89 years were stratified by decades. Changes in splenic surface area and volume were analyzed according to age and sex using measurements obtained from these images with the 3D Slicer software package. **Results:** Splenic volume showed significant differences across age groups in females, whereas no statistically significant age-related difference was observed in males. Splenic surface area differed significantly according to age groups in both sexes. The highest mean splenic volume and surface area were observed in the 19–29 age group in both sexes. The lowest mean values were detected in the 80–89 age group in females and in the 70–79 age group in males. **Conclusions:** Splenic volume is higher in males than in females. A statistically significant decrease in splenic volume with advancing age was observed in females, whereas only a non-significant decreasing trend was noted in males. A marked reduction in splenic volume and surface area was observed particularly after the age of 70 years. This finding is associated with age-related immune decline and functional hyposplenism and carries clinical significance in terms of increased susceptibility to infections and diminished immune response in elderly individuals. Furthermore, splenic shrinkage should be considered in the assessment of hematological reserve and in the differentiation of changes related to chronic diseases.

## 1. Introduction

The spleen plays a critical role in the human body due to its hematologic and immunologic functions [1,2]. Its function as a reservoir for hematopoietic cells and its contribution to the regulation of the hemodynamic response by releasing these cells into the circulation during physiological stress increase the clinical significance of splenic volume. Changes in splenic volume are associated not only with local pathologies but also with systemic diseases and stress responses [3,4]. Therefore, the accurate measurement of splenic volume is valuable both in diagnostic processes and in the clinical follow-up of patients with conditions such as trauma, infection, and hematologic disorders. In the study by Liu et al. (2023) [5], evidence was presented suggesting that preoperative splenic area may serve as a novel, simple, non-invasive, and reproducible radiological biomarker for prognostic assessment and personalized treatment planning in patients with early-stage non-small cell lung cancer.

Splenic size may vary depending on an individual’s height and body weight [6], and may increase in response to systemic infections as well as hepatic, myeloproliferative, or other proliferative disorders [7]. The patient’s clinical history and medical background can provide important insights into the potential causes of changes in splenic volume. However, this variability in splenic volume makes it more challenging to define “normal splenic morphometry” compared with other relatively stable solid organs [6,8].

Organ segmentation refers to the delineation of an organ from the surrounding tissues on clinical imaging. Manual segmentation on cross-sectional CT images is time-consuming, labor-intensive, and subject to interobserver variability [9]. Therefore, its applicability in routine clinical practice is limited. To enhance accuracy and reproducibility, automated and semi-automated segmentation techniques have been developed. Various advanced algorithms for organ extraction from medical images have been described in the literature [10,11,12,13]. One such platform, 3D Slicer, enables the three-dimensional visualization of anatomical structures and automated quantitative analysis using medical imaging data. As a free and open-source software that does not require dedicated hardware, it represents a practical tool for both image analysis and surgical navigation applications [14].

3D Slicer has a wide range of potential applications, including preoperative planning, medical student and trainee education, patient counseling, medical image processing, and the development of artificial intelligence-based algorithms. Its capability to provide three-dimensional visualization and quantitative analysis enables a more detailed and reliable assessment of organ morphometry [15].

In the available literature, no study has systematically investigated the age-related morphometric changes of the spleen across a broad age range using a sex-specific, decade-based classification approach. Therefore, splenic volume and surface area measurements were performed using the 3D Slicer software, and the obtained data were compared across age and sex groups to analyze potential morphometric differences. By identifying age- and sex-related differences, we aimed to contribute to the establishment of more reliable values for splenic morphometry and to provide data that may support clinical decision-making in diagnostic and therapeutic processes.

## 2. Materials and Methods

### 2.1. Study Population and Data Collection

**Figure 1:**
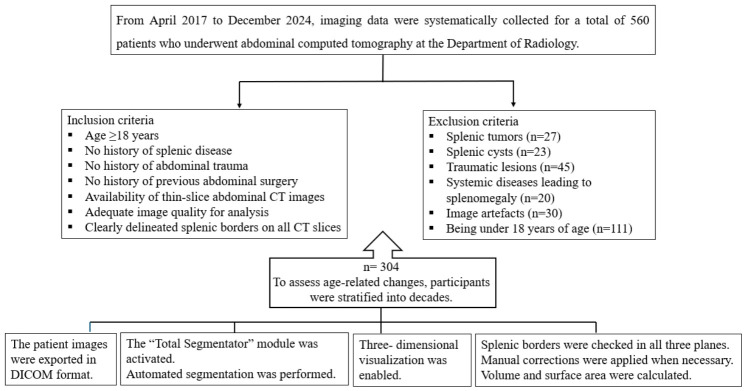
Patient selection flowchart illustrating the inclusion and exclusion criteria for the final study.

A total of 560 patients aged 18 years and older who underwent abdominal computed tomography at the Department of Radiology between 2017 and 2024 were evaluated for splenic morphology. Only individuals without a history of splenic disease, abdominal trauma, chronic liver disease, cirrhosis, hematological disorders, infections, heart failure, malignancy or previous surgery were included. Thin-slice abdominal CT images of these patients were analyzed. Of these, 256 patients were excluded due to splenic tumors, cysts, traumatic lesions, systemic diseases leading to splenomegaly, image artifacts, or being under 18 years of age. Patients younger than 18 years were also excluded because these individuals may not have reached mature organ size [1]. Radiological images were reviewed retrospectively. To assess age-related changes, participants were stratified into decades as follows: 19–29, 30–39, 40–49, 50–59, 60–69, 70–79, and 80–89 years [16] (Figure 1).

### 2.2. Computed Tomography (CT) Protocol

All CT examinations were performed with patients in the supine position using a 128 × 2-detector CT scanner (Somatom Definition Flash, Siemens Healthineers, Forchheim Germany). Scanning was conducted in the craniocaudal direction to include the upper abdomen and pelvic region. Following intravenous contrast administration, images were acquired during the portal venous phase (60–70 s). In the CT protocol, a nonionic iodinated contrast agent with a concentration of 300 mgI/mL was administered via a peripheral vein at a dose of 0.8–1 mL/kg (maximum 100 mL) with an injection rate of 1.8–2.7 mL/s. Imaging parameters included a tube voltage of 120 kVp, a pitch value of 0.8, and the use of effective mAs for tube current modulation. Axial images were reconstructed with a slice thickness of 3 mm.

### 2.3. Morphometric Analysis with 3D Slicer

**Figure 2:**
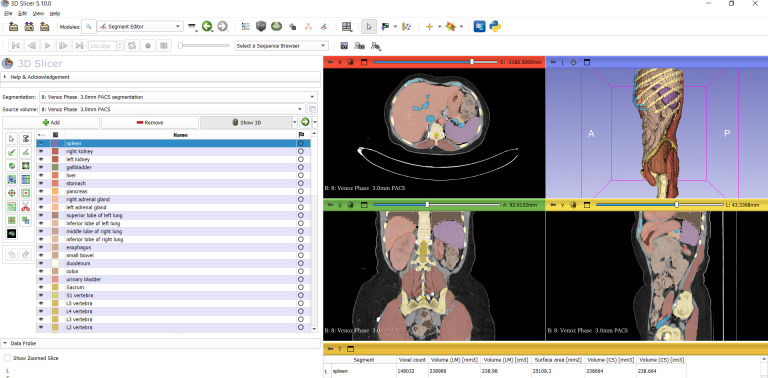
View of spleen in different planes (axial, coronal, sagittal) with 3D Slicer.

**Figure 3:**
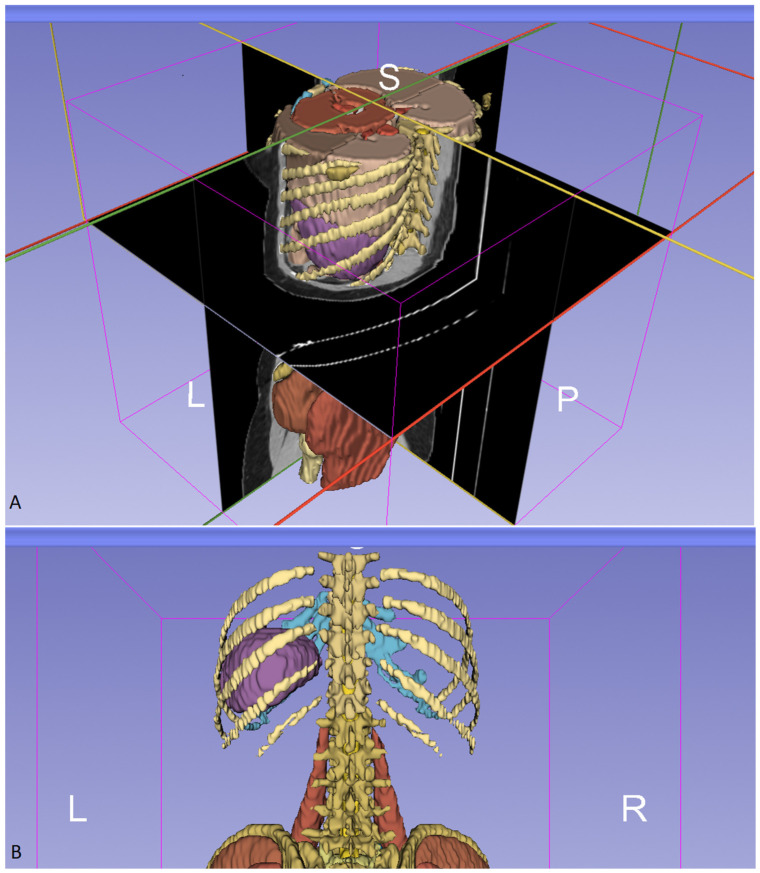
(**A**): Visual of the 3D coordinate system of the spleen. Yellow; sagittal plane, red; horizontal plane, green; coronal plane (**B**): Isolated posterior view of the spleen (L: left, P: posterior, R: right, S: superior).

The patient images obtained in this study were exported in Digital Imaging and Communications in Medicine (DICOM) format. Volumetric analysis of the spleen was performed using the free and open-source software 3D Slicer (version 4.10.2; https://www.slicer.org/) [14]. The imported CT datasets were evaluated in the axial, coronal, and sagittal planes using multiplanar reconstruction. After appropriate image selection, the segmentation process was initiated. The “Total Segmentator” module was activated from the “Modules” menu, and automated segmentation was performed by selecting the “Apply” function. Three-dimensional visualization was enabled using the “Show 3D” option. The spleen was then selected using the “Segment Editor” tool. The anatomical boundaries of the spleen were carefully reviewed in all three planes to ensure complete inclusion of the splenic parenchyma. When necessary, manual corrections were performed to optimize segmentation accuracy. The anatomical boundaries of the spleen, particularly in relation to adjacent structures such as the stomach, left kidney, and splenic hilum, were meticulously evaluated. The absence of any contact or overlap with neighboring structures was confirmed both on the sagittal, coronal, and axial planes (Figure 2) and by inspection of the isolated spleen (Figure 3B). Subsequently, splenic volume and surface area measurement reports were automatically generated using the “Quantification” module [14,15]. All three-dimensional measurements obtained through this method were independently reviewed and confirmed by two experienced investigators before inclusion in the final analysis (Figure 2). Each observer recorded the measurements separately. In cases of discrepancy, a consensus was achieved through joint review. Interobserver agreement was assessed using the intraclass correlation coefficient (ICC). We found excellent reliability as the values were greater than 0.9 (95%) in all parameters.

The spleen was visualized with reference to a three-dimensional (3D) coordinate system. The transverse plane was positioned at the level of the L1 vertebral body, the coronal plane was aligned with the anterior border of the spleen, and the sagittal plane was passed through the level of the vertebral spinous processes (Figure 3).

### 2.4. Statistical Analysis

The data were analyzed using IBM SPSS Statistics Standard Concurrent User Version 26 (IBM Corp., Armonk, NY, USA). Descriptive statistics were presented as number of observations (n), percentage (%), mean, and standard deviation (SD). In the assessment of normality, data were considered to be normally distributed if the absolute skewness value was below ±2.0 and the kurtosis value was below 7.0 [17]. Accordingly, the skewness and kurtosis values of the variables included in the study are presented in Table 1, and the data were found to conform to a normal distribution.

The distribution of sex across age groups was evaluated using chi-square tests (Pearson chi-square or Fisher’s exact test). Comparisons of measurements between sexes according to age groups were performed using the independent samples *t*-test, while comparisons of age groups according to sex were conducted using one-way analysis of variance (ANOVA). For multiple comparisons, post hoc tests were selected according to the results of Levene’s test for homogeneity of variances, using either Tamhane’s T2 or Bonferroni methods as appropriate.

## 3. Results

### 3.1. Demographic Characteristics of the Study Population

In total, 304 individuals were evaluated, comprising 153 females (50.3%) and 151 males (49.7%). The distribution of age groups was similar between sex, with no statistically significant difference observed (χ^2^ = 0.726, *p* = 0.994). These findings indicate a balanced distribution of participants across both sexes and age groups (Table 2).

### 3.2. Age- and Sex-Based Differences in Splenic Volume

Table 3 presents the comparison of splenic volume measurements between sexes across different age groups. Mean splenic volume differed significantly across age groups in females (*p* < 0.001), whereas no significant age-group difference was observed in males (*p* = 0.148). In the overall sample, splenic volume also varied significantly across age groups (*p* = 0.001). In both sexes, the highest mean splenic volume was observed in the 19–29 age group. The lowest mean splenic volume was found in the 80–89 age group in females and in the 70–79 age group in males on a descriptive basis; however, this variation was not statistically significant in males.

When evaluated by sex, males had higher mean splenic volumes than females in all age groups. This difference was statistically significant in the 19–29 years (*p* = 0.027), 40–49 years (*p* = 0.005), 50–59 years (*p* = 0.013), 60–69 years (*p* = 0.044), and 80–89 years (*p* = 0.002) groups. Post hoc comparisons showed that among females, the 19–29 age group had significantly higher splenic volume than the 50–59 and 80–89 age groups, whereas the remaining female age groups did not differ significantly from groups sharing at least one common superscript letter. In the overall sample, the 19–29 age group also had significantly higher splenic volume than the 50–59 and 80–89 age groups, while the other pairwise comparisons were not statistically significant.

**Figure 4:**
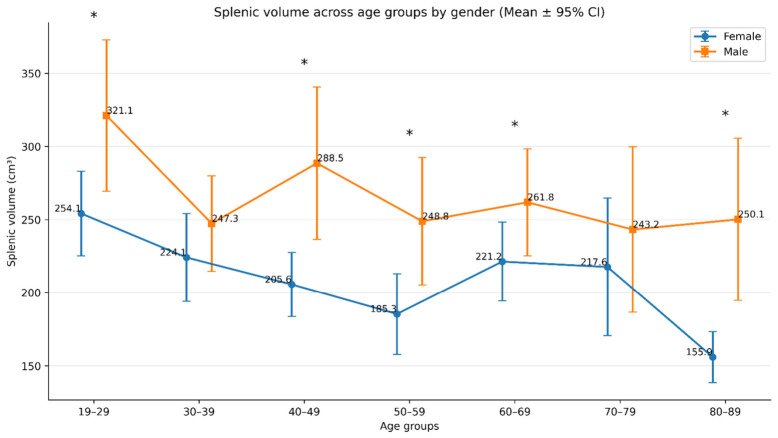
Comparison of splenic volume measurements across age groups by gender. Mean splenic volume across age groups by gender with 95% confidence intervals. Asterisks (*) denote statistically significant sex differences within the same age group (*p* < 0.05).

These findings indicate that splenic volume is associated with sex, with males consistently showing higher values than females, and that age-related differences are evident in females and in the total sample, but not in males (Figure 4).

### 3.3. Age- and Sex-Based Differences in Splenic Surface Area

**Figure 5:**
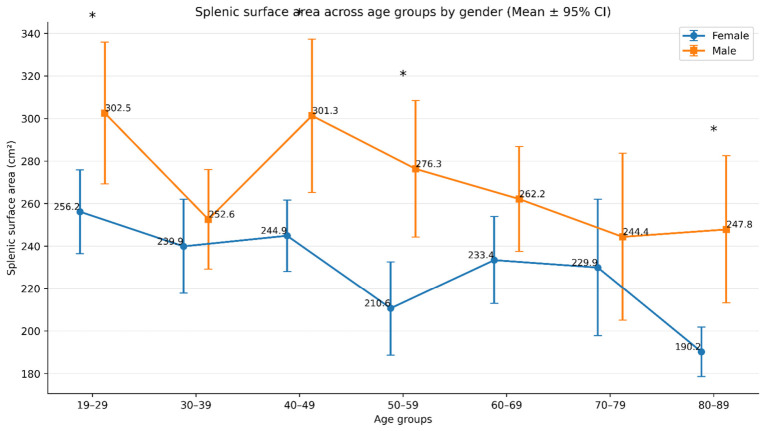
Comparison of splenic surface measurements across age groups by gender. Mean splenic surface area across age groups by gender with 95% confidence intervals. Asterisks (*) denote statistically significant sex differences within the same age group (*p* < 0.05).

A significant age-group difference in splenic surface area was observed in females (*p* < 0.001) and males (*p* = 0.022), and this difference was also significant in the overall sample (*p* < 0.001) (Table 4). Across all age groups, males had higher mean splenic surface area values than females, with statistically significant sex-based differences in the 19–29 (*p* = 0.019), 40–49 (*p* = 0.006), 50–59 (*p* = 0.001), and 80–89 (*p* = 0.002) age groups. Descriptively, the highest mean splenic surface area was observed in the 19–29 age group in both sexes, whereas the lowest mean values were found in the 80–89 age group in females and in the 70–79 age group in males. Post hoc analysis showed that among females, the 80–89 age group had significantly lower splenic surface area than the 19–29, 30–39, 40–49, and 60–69 age groups, whereas the remaining pairwise comparisons were not statistically significant. Among males, although the omnibus ANOVA was significant, Bonferroni-adjusted post hoc comparisons did not identify significant pairwise differences between age groups. In the overall sample, the 80–89 age group had significantly lower splenic surface area than the 19–29 and 40–49 age groups, while the other pairwise comparisons were not statistically significant. Taken together, these findings indicate a descriptively decreasing pattern in splenic surface area with advancing age, although statistically clearer age-related differences were observed in females and in the overall sample than in males (Figure 5).

In both sexes, the highest mean splenic surface area was observed in the 19–29 age group compared with the other age groups. The lowest mean values were found in the 80–89 age group in females and in the 70–79 age group in males. Overall, splenic surface area was higher in younger age groups and showed a decreasing trend with advancing age (Figure 5).

## 4. Discussion

The spleen plays a crucial role in hematologic, immunologic, and reticuloendothelial functions and is therefore of significant importance in the evaluation of systemic diseases. Alterations in splenic volume and surface area have diagnostic and prognostic value in various clinical conditions, including infections, hematologic disorders, portal hypertension, and malignancies [7]. However, the accurate interpretation of pathological splenic enlargement (splenomegaly) or atrophy requires a clear understanding of physiological variations related to age and sex [18]. In the study by Liu et al. (2023) [5], evidence was presented suggesting that preoperative splenic area may serve as a novel, simple, non-invasive, and reproducible radiological biomarker for prognostic assessment and personalized treatment planning in patients with early-stage non-small cell lung cancer. Sex-related differences in splenic surface area are reported to be potentially associated with immune function and tumor biology in the affected patient population. In addition, higher splenic surface area has been observed in male patients compared to females, which has been suggested to be related not only to anthropometric factors, but also to the erythropoiesis-stimulating effects of higher testosterone levels [5].

The assessment of splenic volume is widely adopted for pharmacological and clinical purposes. From a clinical perspective, abnormal splenic volume has significant diagnostic value in the evaluation of liver diseases, hematological malignancies (including myeloproliferative neoplasms and lymphomas), infections, and certain types of anemia [19,20]. From a pharmacological perspective, splenic volume may serve as an indicator in determining the appropriate drug dosage or in evaluating therapeutic efficacy.

An important imaging modality that enables three-dimensional and reliable assessment of splenic volume and surface area is CT. Recent technological advancements in MDCT have improved the diagnostic accuracy of abdominal imaging. Optimal vascular and parenchymal enhancement requires the integrated consideration of patient-related factors, contrast media injection protocols, and scanning parameters [21]. In particular, three-dimensional segmentation techniques provide more accurate morphometric analyses compared with conventional linear measurements. However, in the available literature, no study has been identified that includes a broad age range and evaluates both volume and surface area according to decade-based age classification and sex concurrently. Therefore, the present study aimed to determine age- and sex-related changes in splenic volume and surface area in an adult population and to establish data that may contribute to clinical assessment and decision-making.

Studies evaluating spleen volume in healthy adult populations over the past ten years are summarized in Table 5. The results of studies using CT imaging were generally consistent with each other [22,23,24]. However, studies conducted in the Japanese population reported lower mean spleen volumes [25,26]. This difference suggests that ethnic background may influence splenic morphometry and highlights the importance of considering race- and population-specific values when interpreting splenic measurements. Notably, Takahashi et al. (2019) [25], analyzed pre- and postmortem changes in adult spleen volume using CT. They reported a mean antemortem spleen volume of 98.2 ± 72.5 cm^3^ in healthy adults. This study had the highest mean age and the lowest spleen volume among the studies listed in Table 5. This finding is consistent with our results indicating that splenic volume decreases with advancing age, particularly reflecting the lowest splenic volumes observed in women aged 80–89 years and in men aged 70–79 years.

Splenic volumes reported in studies using ultrasonography (US) have generally been found to be lower compared to CT-based measurements (Table 5) [6,27]. In ultrasonography, volume calculations are mostly based on linear measurements and specific geometric assumptions, which can result in spleen volumes being systematically underestimated relative to CT-based three-dimensional segmentation methods. In the study by Anzoletti et al. (2026) [27], cadaveric spleen volumes were assessed using the water displacement method (immersion in a pre-filled graduated container) (120–580 cm^3^). In the same study, measurements in healthy individuals reported an average volume of 203.02 cm^3^ using the ellipsoid model, and 229.41 cm^3^ using the spherical shell model. These findings clearly demonstrate that the choice of mathematical model directly affects the estimated splenic volume. In our study, the use of voxel-based three-dimensional segmentation allows for a more accurate representation of splenic anatomical boundaries compared to techniques relying on geometric assumptions. This suggests that the volume measurements obtained in our study have a higher methodological accuracy potential. In particular, differences between CT-based three-dimensional volumetric methods and ultrasound-based volume measurement techniques create substantial methodological heterogeneity in terms of measurement principles, spatial resolution, and imaging accuracy, which may lead to variability in the results reported across studies.

In Table 5, all studies that evaluated sex differences reported higher splenic volumes in males compared to females [6,22,23]. This finding was also confirmed in our study. The higher average body surface area and blood volume in males may provide a physiological basis for the observed sex-related differences in splenic volume. In addition, Chiu et al. (2016) [23] found no statistically significant correlation between splenic volume and age (*p* = 0.18). In contrast, in our study, the mean splenic volumes in females differed significantly across age groups (*p* < 0.05; *p* = 0.0001), whereas in males, no significant differences in splenic volume were observed with age (*p* > 0.05; *p* = 0.148). These findings suggest that sex-specific hormonal differences, changes in body composition, or the effects of aging on splenic morphometry may be more pronounced in females.

Studies directly assessing splenic surface area are very limited in the literature. Among these, Sagiroglu et al. (2014) [28] evaluated five neonatal cadavers with a gestational age of 39.7 ± 1.5 weeks and reported the splenic surface area as 32.3 ± 20.6 cm^2^ using the physical section method. On MRI, the surface area was measured as 24.9 ± 15.2 cm^2^, 18.5 ± 5.92 cm^2^, and 24.3 ± 12.7 cm^2^ on the axial, sagittal, and coronal planes, respectively. In adults, Dang et al. (2022) [29] assessed 60 cadavers (30 males and 30 females) aged 16–70 years of Indian origin and reported a mean splenic surface area of 207.59 ± 24.63 cm^2^, ranging from 213–308 cm^2^ in males and 123–202 cm^2^ in females. Both studies are limited by the small sample sizes, which restrict the generalizability of their findings. In our study, the mean splenic surface area was 229.50 ± 50.39 cm^2^ in females, 270.89 ± 74.94 cm^2^ in males, and 250.06 ± 66.96 cm^2^ overall. Our results also revealed statistically significant differences across age groups and between sexes (females: *p* = 0.000; males: *p* = 0.022), demonstrating a declining trend in splenic surface area with advancing age. Furthermore, the use of automated calculation and standardization methods provides our measurements with higher reliability and reproducibility compared to previous studies. Overall, splenic surface area was higher in younger age groups and consistently higher in males across all age ranges. These findings represent a notable contribution to the literature on splenic morphometry.

A major strength of our study is the analysis of CT images in a healthy adult population using three-dimensional segmentation methods. This approach offers several advantages, including the ability to assess the entire organ volume on a voxel-by-voxel basis, eliminate reliance on geometric assumptions, and achieve higher measurement accuracy and reproducibility. Methodological variability observed in ultrasonography and two-dimensional measurement techniques may underlie the differences in splenic volumes reported in the literature. Nonetheless, manual or semi-automated segmentation remains operator-dependent. The literature indicates that fully automated multi-organ segmentation models (e.g., Total Segmentator) provide high accuracy and reproducibility, as well as time efficiency and standardization benefits in large datasets. Validation of such automated approaches in future studies would therefore be highly valuable [15].

Our study evaluated age-related changes in splenic volume and surface area and demonstrated a decline in splenic morphometry with advancing age in both sexes. Notably, a marked reduction in splenic volume and surface area was observed after the age of 70. This finding may be of clinical relevance in the context of immunosenescence and functional hyposplenism in older adults, and it may suggest a potential association with an increased risk of infections caused by encapsulated bacteria, higher sepsis mortality, and reduced vaccine responses. Furthermore, splenic atrophy should be considered in the assessment of conditions characterized by reduced hematologic reserve, diminished extramedullary hematopoietic capacity, or bone marrow failure. It is also important for distinguishing physiological age-related involution from white pulp involution observed in chronic inflammatory and autoimmune diseases. Age-related reductions in splenic perfusion due to atherosclerosis and chronic cardiovascular diseases, as well as recurrent microinfarcts, may further contribute to volume loss. Finally, the pronounced decrease in splenic morphometry may serve as an indicator of frailty and generalized organ atrophy in the geriatric population, highlighting the necessity of using age-specific values in radiological assessments [3,22].

## 5. Conclusions

In conclusion, our study provides a detailed assessment of age- and sex-related changes in splenic volume and surface area in an adult population using voxel-based three-dimensional CT segmentation. Notably, a marked reduction in splenic volume and surface area was observed after the age of 70. We demonstrated that both splenic volume (in female) and surface area decline with advancing age. Sex-specific differences were also evident, with males exhibiting higher splenic volumes and surface areas across all age groups.

The methodological rigor of this study, including automated calculation and standardization, enhances the accuracy, reproducibility, and reliability of the measurements compared to previous studies. Clinically, these findings are relevant to evaluating immunosenescence, functional hyposplenism, and age-related hematologic changes, as well as for distinguishing pathological splenic atrophy from physiological age-related involution [3,22].

Our results provide data for age- and sex-specific splenic morphometry, which may be useful in radiological assessment, clinical decision-making, and future research. Additionally, the observed correlations between splenic morphometry and age underscore the potential role of the spleen as a marker for frailty and generalized organ atrophy in the geriatric population.

This study has several limitations. Its retrospective single-center design limits the generalizability of the findings. In addition, due to the retrospective nature of the dataset, anthropometric variables such as height, weight, body mass index (BMI), and body surface area (BSA) could not be evaluated. Moreover, other systemic factors that may influence spleen size, including liver disease, hematological disorders, and hemodynamic status, were not available and therefore could not be included in the analysis.

## Figures and Tables

**Table 1:** Normality assessment of measurements (*N* = 304).

	Skewness	Kurtosis	Kolmogorov–Smirnov	
			Test	*p*
Volume	1.385	3.030	0.051	0.070
Surface area	0.978	1.508	0.048	0.098

**Table 2:** Distribution of age groups by gender (*N* = 304).

	Gender		Total (n = 304)	*χ* ^2^	*p*
	Female (n = 153)	Male (n = 151)			
	n (*%*)	n (*%*)	n (*%*)		
Age groups				0.726	0.994
19–29	21 (%14)	23 (%15)	44 (%14)		
30–39	22 (%14)	23 (%15)	45 (%15)		
40–49	24 (%16)	25 (%17)	49 (%16)		
50–59	21 (%14)	20 (%13)	41 (%14)		
60–69	24 (%16)	19 (%13)	43 (%14)		
70–79	19 (%12)	19 (%13)	38 (%13)		
80–89	22 (%14)	22 (%14)	44 (%14)		

Chi-square test (χ^2^) was used for categorical variables; descriptive statistics were presented as number (n) and percentage (%).

**Table 3:** Comparison of splenic volume measurements across age groups by gender (cm^3^).

Volume		Gender				Total
		Female	Male			X ± SS
		X ± SS	X ± SS	*t*	*p* (by Gender)	
Age groups	19–29	254.12 ± 63.45 ^B^	321.12 ± 119.80 ^A^	−2.285	0.027 *	289.14 ± 101.79
	30–39	224.08 ± 67.86 ^C^	247.27 ± 75.44 ^BC^	−1.082	0.285	235.93 ± 71.98
	40–49	205.57 ± 52.02 ^C^	288.54 ± 126.39 ^AB^	−2.982	0.005 *	247.90 ± 105.07
	50–59	185.26 ± 60.40 ^C^	248.81 ± 93.22 ^AB^	−2.603	0.013 *	216.26 ± 83.58
	60–69	221.24 ± 64.09 ^C^	261.81 ± 75.82 ^AB^	−1.901	0.044 *	239.17 ± 71.61
	70–79	217.56 ± 97.71 ^C^	243.17 ± 117.51 ^BC^	−0.730	0.470	230.36 ± 107.38
	80–89	155.94 ± 39.18 ^D^	250.12 ± 125.17 ^BC^	−3.368	0.002 *	203.03 ± 103.29
	*F*	5.050	1.611			3.746
	*P*	0.000 * (by age groups)	0.148			**0.001**
Total		208.92 ± 69.67	267.28 ± 109.01	−5.569	**0.000**	237.91 ± 95.77

* *p* < 0.05 was considered statistically significant. Paired-samples *t*-test (t) and one-way ANOVA (F) were used for statistical comparisons. Descriptive statistics were presented as mean (X) and standard deviation (SD). Values shown in bold indicate statistically significant differences (*p* < 0.05). A > B > C > D: Differences between groups indicated by different letters within the same row or column are statistically significant (LSD test, *p* < 0.05).

**Table 4:** Comparison of splenic area measurements across age groups by gender.

Surface Area		Gender				
		Female	Male			Total
		X ± SS	X ± SS	*t*	*p*	X ± SS
Age groups	19–29	256.19 ± 43.23 ^B^	302.54 ± 77.02 ^A^	−2.429	0.019 *	280.42 ± 66.73
	30–39	239.90 ± 49.84 ^B^	252.58 ± 54.06 ^B^	−0.817	0.419	246.38 ± 51.84
	40–49	244.89 ± 39.86 ^B^	301.32 ± 87.31 ^A^	−2.890	0.006 *	273.68 ± 73.38
	50–59	210.63 ± 48.17 ^C^	276.32 ± 68.50 ^B^	−3.566	0.001 *	242.67 ± 67.04
	60–69	233.41 ± 48.61 ^B^	262.16 ± 51.08 ^AB^	−1.884	0.067	246.11 ± 51.19
	70–79	229.91 ± 66.64 ^B^	244.36 ± 81.49 ^B^	−0.598	0.553	237.14 ± 73.79
	80–89	190.21 ± 26.14 ^D^	247.84 ± 78.06 ^B^	−3.283	0.002 *	219.03 ± 64.49
	*F*	4.911	2.554			4.802
	*P*	0.000 *	0.022 *			**0.000**
Total		229.50 ± 50.39	270.89 ± 74.94	−5.657	**0.000**	250.06 ± 66.96

* *p* < 0.05 was considered statistically significant. Paired-samples *t*-test (t) and one-way ANOVA (F) were used for statistical comparisons. Descriptive statistics were presented as mean (X) and standard deviation (SD). Values shown in bold indicate statistically significant differences (*p* < 0.05). A > B > C: Differences between groups indicated by different letters within the same row or column are statistically significant (LSD test, *p* < 0.05).

**Table 5:** A comparison of spleen volume among various studies (cm^3^).

	Method	Population	Age	N (F-M)	Female	Male	Total
Cruz-Romero et al. (2016) [22]	CT	American	48.6 ± 18 years	101 (69–32)	216.6 ± 104.5 cm^3^	304.3 ± 119.6 cm^3^	244 ± 116.4 cm^3^ Min: 72 cm^3^ Max: 590 cm^3^
Chow et al. (2016) [6]	US	German	31 years (range: 18–55 years)	1200	134 cm^3^	179 cm^3^	166 cm^3^
Chiu et al. (2016) [23]	CT	American	65.9 ± 14.5 years	82 (33–49)	185 ± 123 cm^3^	251 ± 138 cm^3^	224.5 ± 135.5 cm^3^
Takahashi et al. (2019) [25]	CT	Japanese	75 years (range: 34–92 years)	44			98.2 ± 72.5 cm^3^
Kim et al. (2021) [24]	CT	Korean	30 ± 9.334 years	472			Min: 81.1 cm^3^ Max: 322.0 cm^3^
Kawashima et al. (2022) [26]	CT	Japanese	44.7 ± 4.9 years	19 (13–6)			134.6 ± 65.2 cm^3^
Anzoletti et al. (2026) [27]	Cadaver: graduated container HI: US	Cadaver: Unspecified HI: Italian	Cadaver: 66.8 years (42–84 years) HI: 60 years and over	Cadaver: 15 (4–11) HI: 101			Cadaver: Min: 120 cm^3^ Max: 580 cm^3^ HI: Ellipsoid volume: 203.02 cm^3^ Spherical Shell volume: 229.41 cm^3^
Present Study	CT images using the 3D Slicer software	Turkish	Range: 19–89 years	304 (153–151)	208.92 ± 69.67 cm^3^	267.28 ± 109.01 cm^3^	237.91 ± 95.77 cm^3^

CT: computerized tomography, F: female, HI: healthy individual, M: male, US: ultrasound.

## Data Availability

The data that support the findings of this study are available from the corresponding author upon reasonable request.

## References

[1] Geraghty E.M., Boone J.M., McGahan J.P., Jain K. (2004). Normal organ volume assessment from abdominal CT. Abdom. Imaging.

[2] Harris A., Kamishima T., Hao H.Y., Kato F., Omatsu T., Onodera Y., Terae S., Shirato H. (2010). Splenic volume measurements on computed tomography utilizing automatically contouring software and its relationship with age, gender, and anthropometric parameters. Eur. J. Radiol..

[3] Kaneko J., Sugawara Y., Matsui Y., Ohkubo T., Makuuchi M. (2002). Normal splenic volume in adults by computed tomography. Hepatogastroenterology.

[4] Kosmiski L., Schmiege S.J., Mascolo M., Gaudiani J., Mehler P.S. (2014). Chronic starvation secondary to anorexia nervosa is associated with an adaptive suppression of resting energy expenditure. J. Clin. Endocrinol. Metab..

[5] Liu M., Yan G., Li Y., You R., Liu L., Zhang D., Li Z. (2023). Preoperative splenic area as a prognostic biomarker of early-stage non-small cell lung cancer. Cancer Imaging.

[6] Chow K.U., Luxembourg B., Seifried E., Bonig H. (2016). Spleen size is significantly influenced by body height and sex: Establishment of normal values for spleen size at US with a cohort of 1200 healthy individuals. Radiology.

[7] Suttorp M., Classen C.F. (2021). Splenomegaly in children and adolescents. Front. Pediatr..

[8] Chaware P., Belsare S., Kulkarni Y., Pandit S., Ughade J. (2012). The morphological variations of the human spleen. J. Clin. Diagn. Res..

[9] Nuffer Z., Marini T., Rupasov A., Kwak S., Bhatt S. (2017). The best single measurement for assessing splenomegaly in patients with cirrhotic liver morphology. Acad. Radiol..

[10] Xiong W., Ong S.H., Tian Q., Xu G., Zhou J., Liu J., Venkatash S.K. (2009). Construction of a linear unbiased diffeomorphic probabilistic liver atlas from CT images. 2009 16th IEEE International Conference on Image Processing (ICIP).

[11] Karasev P., Kolesov I., Fritscher K., Vela P., Mitchell P., Tannenbaum A. (2013). Interactive medical image segmentation using PDE control of active contours. IEEE Trans. Med. Imaging.

[12] Umetsu S., Shimizu A., Watanabe H., Kobatake H., Nawano S. (2014). An automated segmentation algorithm for CT volumes of livers with atypical shapes and large pathological lesions. IEICE Trans. Inf. Syst..

[13] Juszczyk J., Pietka E., Pyciński B. (2015). Granular computing in model based abdominal organs detection. Comput. Med. Imaging Graph..

[14] Fedorov A., Beichel R., Kalpathy-Cramer J., Finet J., Fillion-Robin J.-C., Pujol S., Bauer C., Jennings D., Fennessy F., Sonka M. (2012). 3D slicer as an image computing platform for the quantitative imaging network. Magn. Reson. Imaging.

[15] Wasserthal J., Breit H.C., Meyer M.T., Pradella M., Hinck D., Sauter A.W., Segeroth M. (2023). Total Segmentator: Robust segmentation of 104 anatomic structures in CT images. Radiol. Artif. Intell..

[16] Caglar V., Songur A., Yagmurca M., Acar M., Toktas M., Gonul Y. (2012). Age-related volumetric changes in pancreas: A stereological study on computed tomography. Surg. Radiol. Anat..

[17] Kim H.-Y. (2013). Statistical notes for clinical researchers: Assessing normal distribution (2) usings kewness and kurtosis. Restor. Dent. Endod..

[18] Wu W.C., Chiou Y.Y., Hung H.H., Kao W.Y., Chou Y.H., Su C.W., Wu J.C., Huo T.I., Huang Y.H., Lee K.C. (2012). Prognostic significance of computed tomography scan-derived splenic volume in the patocellular carcinoma treated with radiofrequen cy ablation. J. Clin. Gastroenterol..

[19] Paley M.R., Ros P.R. (2002). Imaging of spleen disorders. The Complete Spleen: Structure, Function, and Clinical Disorders.

[20] Moon H., Huo Y., Abramson R.G., Peters R.A., Assad A., Moyo T.K., Landman B.A. (2019). Acceleration of spleen segmentation with end-to-end deep learning method and automated pipeline. Comput. Biol. Med..

[21] Rengo M., Bellini D., De Cecco C.N., Osimani M., Vecchietti F., Caruso D., Laghi A. (2011). The optimal contrast media policy in CT of the liver. Part I: Technical notes. Acta Radiol..

[22] Cruz-Romero C., Agarwal S., Abujudeh H.H., Thrall J., Hahn P.F. (2016). Spleen volume on CT and the effect of abdominal trauma. Emerg. Radiol..

[23] Chiu N.L., Kaiser B., Nguyen Y.V., Welbourne S., Lall C., Cramer S.C. (2016). The volume of the spleen and its correlates after acute stroke. J. Stroke Cerebrovasc. Dis..

[24] Kim D.W., Ha J., Lee S.S., Kwon J.H., Kim N.Y., Sung Y.S., Kang B.K. (2021). Population-based and personalized reference intervals for liver and spleen volumes in healthy individuals and those with viral hepatitis. Radiology.

[25] Takahashi N., Yajima K., Otaki M., Yoshikawa Y., Ishihara A., Sato Y., Takatsuka H. (2019). Postmortem volume change of the spleen and kidney on early postmortem computed tomography: Comparison with antemortem computed tomography. Jpn. J. Radiol..

[26] Kawashima K., Onizawa M., Fujiwara T., Gunji N., Imamura H., Katakura K., Ohira H. (2022). Evaluation of the relationship between the spleen volume and the disease activity in ulcerative colitis and Crohn disease. Medicine.

[27] Anzoletti N., Rotunno L., Pitocco A., D’Alessandro P., Cocco G., Di Lorito A., Schiavone C. (2026). Ellipsoid vs spherical cap: A new approach to splenic volumetry validated on cadavers. J. Ultrasound.

[28] Sagiroglu A., Acer N., Ertekin T., Kurtoglu E., Coskun A., Yildirim A., Zararsiz G. (2014). Estimation of spleen volume and surface area of the newborns’ cadaveric spleen using stereological methods. Folia Morphol..

[29] Dang B., Gupta S., Batra R., Bokariya P., Malik V.S., Rohila J. (2022). Correlation of Morphometric Parameters of Spleen and Height of the Deceased: A Cross-sectional Study in North Indian Population. J. Clin. Diagn. Res..

